# *Rb1* loss modifies but does not initiate alveolar rhabdomyosarcoma

**DOI:** 10.1186/2044-5040-3-27

**Published:** 2013-11-25

**Authors:** Ken Kikuchi, Eri Taniguchi, Hung-I Harry Chen, Matthew N Svalina, Jinu Abraham, Elaine T Huang, Koichi Nishijo, Sean Davis, Christopher Louden, Lee Ann Zarzabal, Olivia Recht, Ayeza Bajwa, Noah Berlow, Mònica Suelves, Sherrie L Perkins, Paul S Meltzer, Atiya Mansoor, Joel E Michalek, Yidong Chen, Brian P Rubin, Charles Keller

**Affiliations:** 1 Department of Pediatrics, Pediatric Cancer Biology Program, Papé Family Pediatric Research Institute, Portland, OR 97239, USA; 2 Departments of Epidemiology & Biostatistics, Greehey Children’s Cancer Research Institute, University of Texas Health Science Center, San Antonio, TX 78229, USA; 3 Oncogenomics Section, Pediatric Oncology Branch, Advanced Technology Center, National Cancer Institute, Gaithersburg, MD 20877, USA; 4 Department of Pathology, Oregon Health & Science University, Portland, OR 97239, USA; 5 Institut de Medicina Predictiva i Personalitzada del Càncer, Ctra. de Can Ruti, Barcelona 08916, Spain; 6 ARUP Laboratories and Department of Pathology, University of Utah, Salt Lake City, UT 84112, USA; 7 Departments of Anatomic Pathology and Molecular Genetics, Taussig Cancer Center and Lerner Research Institute, Cleveland Clinic Foundation, Cleveland, OH 44195, USA

**Keywords:** Alveolar rhabdomyosarcoma, Disease modifier, Sarcoma, Rb1, Spindle cell, Retinoblastoma

## Abstract

**Background:**

Alveolar rhabdomyosarcoma (aRMS) is a myogenic childhood sarcoma frequently associated with a translocation-mediated fusion gene, *Pax3:Foxo1a.*

**Methods:**

We investigated the complementary role of *Rb1* loss in aRMS tumor initiation and progression using conditional mouse models.

**Results:**

*Rb1* loss was not a necessary and sufficient mutational event for rhabdomyosarcomagenesis, nor a strong cooperative initiating mutation. Instead, *Rb1* loss was a modifier of progression and increased anaplasia and pleomorphism. Whereas *Pax3:Foxo1a* expression was unaltered, biomarkers of aRMS versus embryonal rhabdomyosarcoma were both increased, questioning whether these diagnostic markers are reliable in the context of *Rb1* loss. Genome-wide gene expression in *Pax3:Foxo1a,Rb1* tumors more closely approximated aRMS than embryonal rhabdomyosarcoma. Intrinsic loss of pRb function in aRMS was evidenced by insensitivity to a Cdk4/6 inhibitor regardless of whether *Rb1* was intact or null. This loss of function could be attributed to low baseline *Rb1*, pRb and phospho-pRb expression in aRMS tumors for which the *Rb1* locus was intact. *Pax3:Foxo1a* RNA interference did not increase pRb or improve Cdk inhibitor sensitivity. Human aRMS shared the feature of low and/or heterogeneous tumor cell pRb expression.

**Conclusions:**

*Rb1* loss from an already low pRb baseline is a significant disease modifier, raising the possibility that some cases of pleomorphic rhabdomyosarcoma may in fact be Pax3:Foxo1a-expressing aRMS with *Rb1* or pRb loss of function.

## Background

The pediatric and young adult tumor, rhabdomyosarcoma (RMS), is increasingly being understood to represent a spectrum of diseases that are distinguished not only by histological appearance but also by mutational profile and cell of origin [1-3]. Two major subtypes of RMS exist, alveolar rhabdomyosarcoma (aRMS) and embryonal rhabdomyosarcoma (eRMS) [4]. aRMS is commonly associated with a translocation-mediated *PAX3:FOXO1A* fusion gene [4], whereas the best described initiating mutation in eRMS is *p53* loss [1]. The rarer anaplastic variant of RMS is incompletely understood, although the adult pleomorphic RMS variant is now thought to be often driven by Ras [5].

A high frequency of retinoblastoma (*Rb1*) gene mutation has been reported in a subset of human eRMS [6], and we previously reported that *Rb1* nullizygosity in combination with other mutations may lead to loss of differentiation in eRMS and spindle cell sarcomas [1]. However, the role of *Rb1* loss in aRMS remains controversial [6,7]. In this study, we employ conditional mouse genetics to define the role of *Rb1* in the initiation and progression of aRMS.

The primary aim of this study was to determine the role of *Rb1* loss in tumor initiation and progression using conditional genetic mouse models of aRMS. We hypothesized that *Rb1* plays a critical role in tumor initiation, but instead identified *Rb1* loss as a disease modifier resulting in not only anaplasia but also a switch from aRMS to pleomorphic RMS identity. Our studies also point to an inherently low expression of pRb in aRMS, even when the *Rb1* locus is intact.

## Methods

### Mice

All animal procedures were conducted in accordance with the Guidelines for the Care and Use of Laboratory Animals and were approved by the Institutional Animal Care and Use Committee at the University of Texas Health Science Center at San Antonio or the Oregon Health & Science University. The *Myf6Cre*, conditional *Pax3:Foxo1a*, conditional *p53*, and conditional *Rb1* mouse lines and corresponding genotyping protocols have been described previously [2,8-12]. Tumor-prone mice were visually inspected every 2 days for tumors because of the fulminant onset in these models. Tumor staging was based upon a previously described adaptation of the Intergroup Rhabdomyosarcoma Study Group staging system [1].

### Human subjects

The Oregon Health & Science University institutional review board has made a determination that the use of de-identified tumor samples from the Nationwide Children’s Hospital Biopathology Center or Children’s Oncology Group Biorepository (both sources that consent patients for research tissues directly) is not human subject research because these activities do not meet the definition of human subject per 45 CFR 46.102(f).

### Survival analysis

Kaplan–Meier survival analysis of the mice was performed with the endpoint being the development of RMS. The log-rank test was utilized to determine the statistical significance (*P* < 0.05). Both analyses were performed with Systat12 software (Systat Software Inc., Chicago, IL, USA).

### RNA isolation and quantitative reverse transcription-polymerase chain reaction (qRT-PCR)

**Figure 1:**
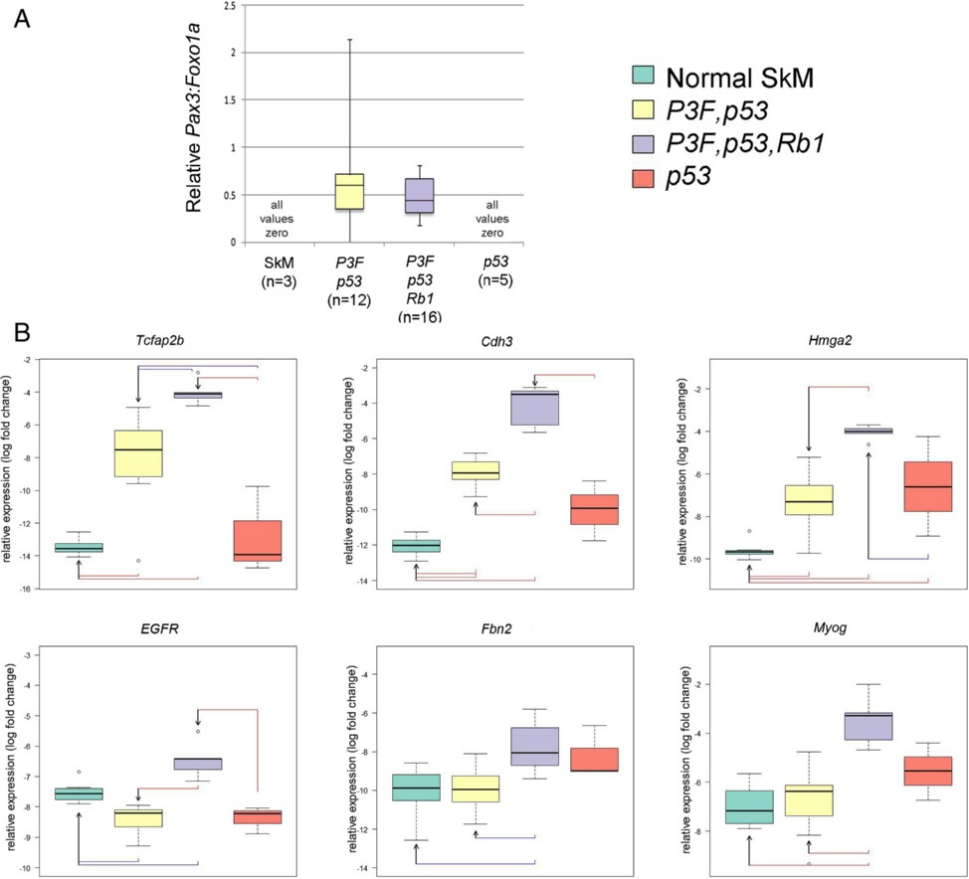
**Addition of *Rb1 *inactivation to *Pax3:Foxo1a *activation and *p53 *deletion changes gene expression features for tumors in the *Myf6cre *lineage. (A)** qRT-PCR of *Pax3:Foxo1a* shows similar expression level in strains with *Pax3:Foxo1a,p53* versus *Pax3:Foxo1a,p53,Rb1* mice. **(B)** Expression of tumor subtype-specific markers in *Pax3:Foxo1a,p53* versus *Pax3:Foxo1a,p53,Rb1* versus *p53* mice.

RNA was isolated from mouse tumors and wildtype *vastus lateralis* skeletal muscle using Trizol (Invitrogen, Carlsbad, CA, USA) following the manufacturer’s instructions. RNA was then processed by RNAeasy-Mini Kit (Qiagen, Valencia, CA, USA) and was reverse transcribed using a first-strand cDNA synthesis kit (Fermentas, Ontario, Canada). For Figure 1A, qRT-PCR analyses were performed on an ABI7700 instrument (PE Applied Biosystems, Foster City, CA, USA) by a Taqman assay for mouse *Pax3:Foxo1a* expression. The mean of three experimental replicates per specimen was used to calculate the ratio of gene of interest/*Gapdh* expression for the Taqman assay, as described previously [11]. For Figure 1B, qRT-PCR was performed using a standard 96-well assay or custom Format-24 Taqman arrays (ABI and Assuragen, Austin, TX, USA) using mouse or human *GAPDH* as a control for relative gene expression, and 18S RNA as a quality control. Statistical considerations for this format assay have been previously described [1]. Probesets for mouse samples were *18S-Hs99999901_s1*, *GAPDH_Mm99999915_g1*, *myog_Mm00446194_m1*, *Cdh3_Mm01249209_m1*, *MYCN_Mm00627179_m1*, *EGFR_Mm00433023_m1*, *Fbn2_Mm00515742_m1*, *tcfap2b_Mm00493468_m1*, *Hmga2_Mm04183367_g1* and *Rb1_Mm00485586_m1*.

### Histology and immunohistochemistry

Tissues fixed in 10% buffered formalin were paraffin-embedded and sectioned at 3.5 μm thickness. Paraffin sections were stained with hematoxylin and eosin or by Gomori Trichrome. For MyoD and Myogenin immunohistochemistry, staining was performed using the M.O.M. Immunodection Kit Staining Procedure (Vector Laboratories, Burlingame, CA, USA) following the manufacturer’s instructions using antigen unmasking. The myogenin monoclonal primary antibody (5D7 supernatant; Developmental Hybridoma Studies Bank, Iowa City, IA, USA) was used at a concentration of 1:50. The Desmin monoclonal primary antibody (Sigma Aldrich, St Louis, MO, USA) was used at a concentration of 1:200. For histology, we evaluated 24 *Pax3:Foxo1a,p53,Rb1* tumors, six *Myf6Cre,Pax3:Foxo1a,Rb1* tumors and two *Myf6Cre,Rb1* tumors (as stated in Results and detailed in our prior publication [13], most *Myf6Cre,Rb1* mice develop pituitary adenomas well ahead of sarcoma development). For the tissue microarray obtained from the Children’s Oncology Group Biorepository, the section was pretreated with Cell Conditioning 1 for 64 minutes as antigen retrieval and then stained with rabbit polyclonal anti-phospho-pRb (Ser807/811, catalogue number9308; Cell Signaling, Danvers, MA, USA) at a dilution of 1:200 followed by staining on a Ventana ES auto stainer (Ventana, Tucson, AZ, USA) and 3,3′-diaminobenzidine detection.

### Cell culture

To establish primary tumor cell cultures, mouse-derived tumors were digested with 1% collagenase IV (Sigma Aldrich) overnight, rinsed with phosphate-buffered saline, and then plated on 10 cm dishes. Cells were cultured in Dulbecco’s modified Eagle’s media (DMEM; Sigma Aldrich) supplemented with 10% fetal bovine serum. The C2C12 mouse myoblast cell line was purchased from ATCC (Manassas, VA, USA) and maintained in the same culture conditions as primary tumor cell cultures.

### Cell viability screens

**Figure 2:**
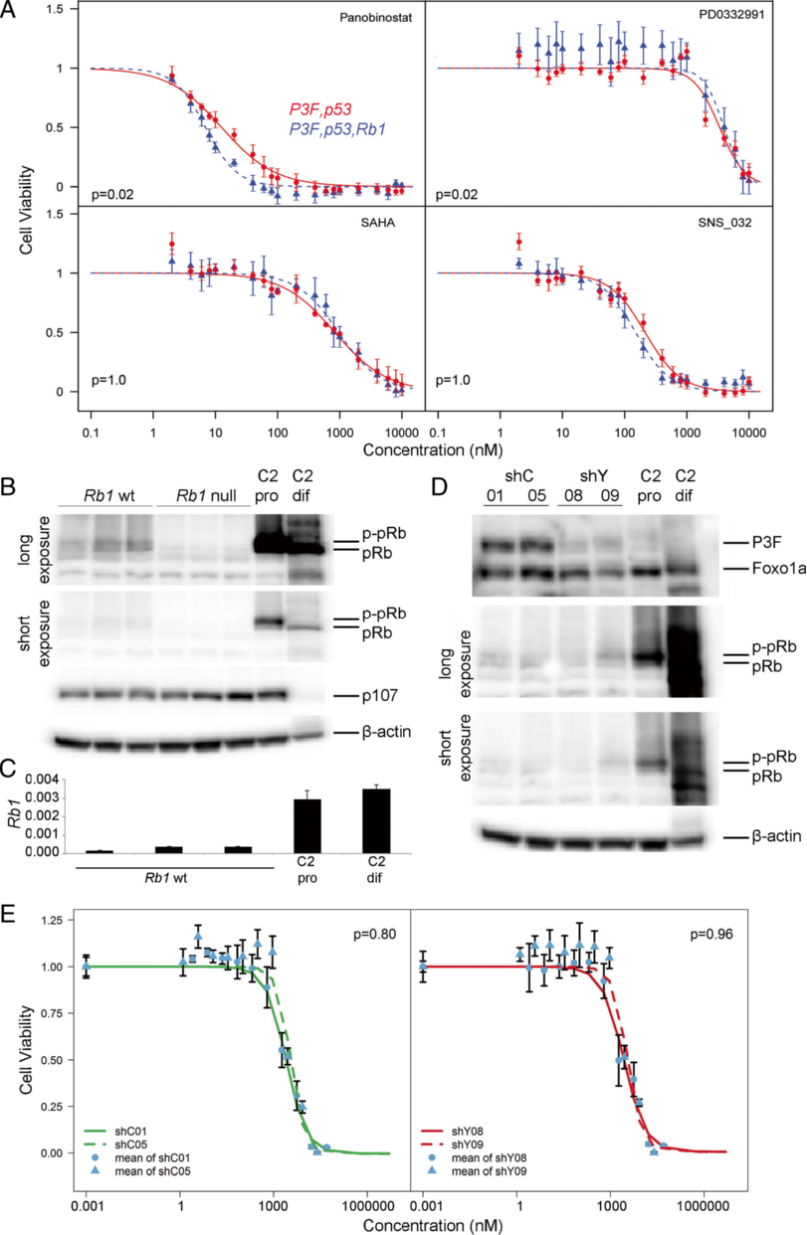
**Sensitivity to CDK4/6 inhibitors is not determined by *Rb1 *status or *Pax3:Foxo1a *expression. (A)** Median inhibitory concentration (IC_50_) determinations by cell viability assays for *Pax3:Foxo1a,p53* (*n* = 3) and *Pax3:Foxo1a,p53,Rb1* (*n* = 3) RMS primary cell cultures treated with Panobinostat, PD0332991, SAHA and SNS-032. *P* values based on a linear model of cell viability in terms of genotype, concentration and the genotype by concentration interaction with Bonferroni multiple testing correction. IC_50_ of PD0332991 was approximately 3 μM for both groups. Error bars, mean ± 1 standard error of the mean. **(B)** Western blotting for *Rb1* wildtype primary tumor cell cultures (*n* = 3) and *Rb1* null RMS primary tumor cell cultures (*n* = 3). C2, C2C12 mouse myoblasts; pro, proliferating culture conditions; dif, differentiation culture conditions. pRb and phospho-pRb (p-Rb) are distinguished by mobility on a 5% gel using a single antibody. Whereas pRb expression is diminished in RMS cell cultures relative to C2C12 proliferating myoblasts, p107 expression is comparable. **(C)** Reduced relative expression levels of *Rb1* by qRT-PCR in *Rb1* wildtype aRMS primary cell cultures relative to C2 pro or dif. 3 independent aRMS primary cultures. All measurements performed in triplicate. *P* ≤0.035 for comparisons of aRMS cultures with either C2 pro or dif. **(D)** Two independent shRNA clones for eYFP knockdown (shY08, shC09; also achieves Pax3:Foxo1a knockdown, see Results) were compared with shRNA controls (shC01, shC05) for expression of pRb, which was unaltered. P3F, Pax3:Foxo1a. **(E)** Insensitivity to PD0332991 was not improved in Pax3:Foxo1a knockdown tumor cell culture clones (IC_50_ of all clones ~2.75 μM). Specifically, shC01 and shC05 independent clones did not differ significantly, shY08 and shY09 independent clones did not differ significantly, nor did the shC versus shY clones differ significantly with regard to mean cell viability.

Mouse-derived primary cell cultures at passage ≤5 plated into 96-well plates using DMEM culture medium supplemented with 10% fetal bovine serum. After 12-hour incubation, vehicle or drug was applied to the cells over a range of concentrations from 0.1 to 10,000 nM in triplicate. Panibinostat, PD0332991, SAHA and SNS-032 were purchased from a commercial source (Selleckchem, Houston, TX, USA). Following 72-hour incubation, an MTS viability assay was performed according to the manufacturer’s instructions (CellTiter 96^®^ AQueous MTS Reagent; Promega, Madison, WI, USA) and quantified using a Synergy 2 Multi-Mode Microplate Reader (Biotek, Winooski, VT, USA) and subsequently analyzed using Microsoft Excel. For Figure 2E, group contrasts (shC01 (*n* = 14) with shC05 (*n* = 14), and shY08 (*n* = 14) with shY09 (*n* = 14)) with regard to mean cell viability were carried out with analyses of covariance of log cell viability in terms of log concentration and group; four data points with negative cell viability for shC01 (*n* = 2) and shC05 (*n* = 2) were removed prior to analysis. After pooling shC01 with shC05 and shY08 with shY09, and removing the four data points with negative cell viability, the resulting two groups (shC, *n* = 24; and shY, *n* = 28) were contrasted with regard to mean cell viability with a similar analysis of covariance model in log units. All statistical testing was two-sided with a significance level of 5%.

### Immunoblotting

*Rb1* wildtype aRMS primary tumor cell cultures, *Rb1* null aRMS primary tumor cell cultures and C2C12 cells were cultured in DMEM with 10% fetal bovine serum and lysed in radioimmunoprecipitation assay buffer containing both protease and phosphatase inhibitor (Sigma-Aldrich) at the proliferation stage (50 to 70% confluency). C2C12 cells were cultured in DMEM with 2% house serum for 7 days and lysed in radioimmunoprecipitation assay buffer as for C2C12 differentiation. The lysates were homogenized and centrifuged at 8,000 × *g* for 10 minutes. The resulting supernatants were used for immunoblot analysis by mouse anti-β-actin (catalogue number A1978; Sigma), mouse anti-pRb (catalogue number 554136; BD Biosciences, San Jose, CA, USA), rabbit anti-p107 (catalogue number sc-318; Santa Cruz Biotechnology, Dallas, TX, USA) and goat anti-FKHR (catalogue number sc-9808; Santa Cruz Biotechnology). For Figure 2B,D, β-actin was run as a separate blot (using the same amount of protein loaded per well as the pRb/p107 blot) rather than stripping because achieving separation of pRb and phospho-pRb on a 5% gel required running β-actin off the gel.

### Generation of shRNA tumor cell culture clones

To establish shRNA knockdown clones of primary tumor cell cultures, we used MISSION^®^ pLKO.1-puro *eGFP* shRNA Control Transduction Particles (catalogue number SHC005V; Sigma Aldrich) for *Pax3:Foxo1a* knockdown and MISSION^®^ pLKO.1-puro Non-Mammalian shRNA Control Transduction Particles (catalogue number SHC002V; Sigma Aldrich) as the control, respectively. shRNA transfections and clonal selection were carried out according to the manufacturer’s recommended procedures. Mouse RMS primary cell cultures were plated at 1.8 × 10^6^ cells per 150 mm dish. After 24 hours, hexadimethrine bromide was added (8 μg/ml, catalogue number H9268; Sigma Aldrich), followed by each particle solution (Multiplicity of Infection 0.5). After another 24 hours, media were removed and fresh media were added. The following day, puromycin was added (5 μg/ml, catalogue number P8833; Sigma Aldrich). Puromycin-resistant clones were selected by cloning rings at day 14 (shC) and day 17 (shY), with continuous puromycin selection at all times.

### Principal component analysis gene selection and microarray analysis

Gene expression analysis was performed using Illumina Mouse Ref-8 Beadchip v1. Microarray datasets were obtained from the GEO database [GEO:GSE22520] from our previous study [1]. We employed similar methods for microarray data analysis and the principal component analysis (PCA) described by Rubin and colleagues [1]. Briefly, we first performed rank invariant set normalization on mouse gene expression data, and then selected 12,370 probes out of 24,613 probes from Mouse Ref-8 beadchip with average log_2_ intensity >6 and standard deviation >0.1 over 25 samples. We also derived four gene sets for PCA from different studies (Table 1) to show the relevance of aRMS-like and eRMS-like tumors between mouse and human. All four signature gene sets are first mapped from human to mouse gene symbols via homolog utility at MammalHom (Table 1), and then map to microarray probes if the corresponding probes exist. The reduction of gene count was due simply to the microarray platform difference. These gene lists are presented in Additional file 1.

**Table 1:** Principal component analysis from different 4 studies

**Dataset**	**Number of signature genes**	**Number of genes mapped to microarray**	**Reference**
Differentially expressed genes in fusion-positive aRMS vs. fusion-negative eRMS	121	83	Laé and colleagues [19]
Genes differentially expressed between PAX-FOXO1A and fusion-negative RMS cell lines	79	55	Davicioni and colleagues [14]
Williamson F1/F2 metagenes	45	32	Williamson and colleagues [20]
Genes conserved in mouse and human Pax3:Foxo1a-positive aRMS	58	47	Nishijo and colleagues [11]

All human gene symbols were translated to their homologues in mouse using MammalHom (http://depts.washington.edu/l2l/mammalhom.html) and then mapped to the microarray annotation. aRMS, alveolar rhabdomyosarcoma; eRMS, embryonal rhabdomyosarcoma; RMS, rhabdomyosarcoma.

Microarray datasets were obtained from the GEO database [GEO:GSE22520] from our previous study [1]. We employed similar methods for microarray data analysis and the PCA described by Rubin and colleagues [1]. Briefly, we first performed rank invariant set normalization on mouse gene expression data and applied PCA to the mouse data, respectively, using the selected genes listed in four aRMS versus eRMS signature gene sets. PCA was performed using the MATLAB Bioinformatics toolbox (Mathworks, Natick, MA, USA).

For the comparison between six samples of *Pax3:Foxo1a,p53* tumors (aRMS) and six samples of *Pax3:Foxo1a,p53,Rb1* tumors (other RMS), the normalized expression data were applied to a *t* test and differential expressed genes between aRMS and other RMS tumors were identified with the criterion of fold-change >2 and *P* < 0.05. All bioinformatics tasks were performed with MATLAB/Bioinformatics Toolbox, unless otherwise noted.

## Results

### Rb1 inactivation in combination with Pax3:Foxo1a activation and p53 inactivation causes highly aggressive rhabdomyosarcoma tumors

**Figure 3:**
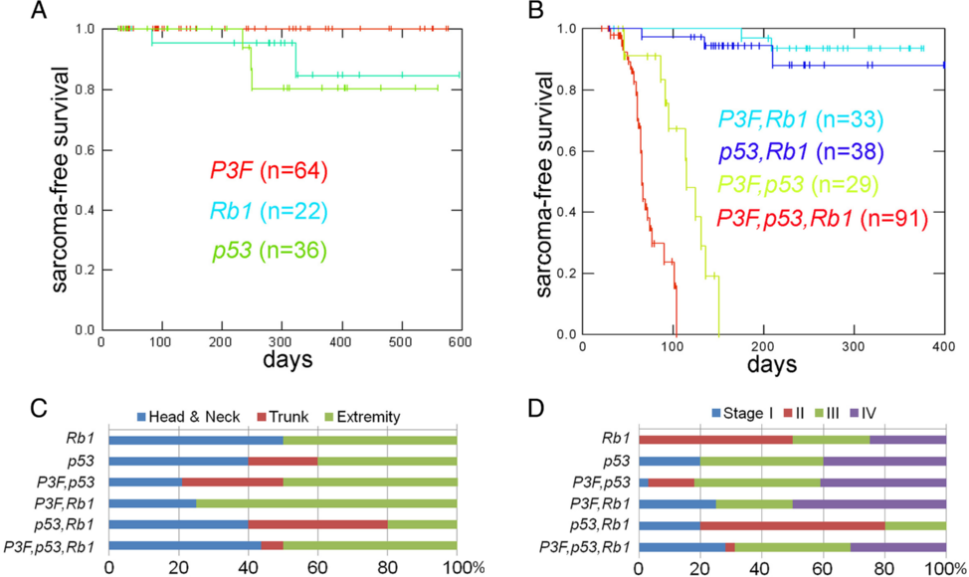
***Rb1 *inactivation decreases latency of rhabdomyosarcoma in with the context of dual *Pax3:Foxo1a *activation and *p53 *inactivation. (A)** Kaplan–Meier survival curve of RMS-free survival in mice with *Rb1* inactivation, *p53* inactivation or *Pax3:Foxo1a* activation in the *Myf6cre* lineage. Any of these genetic events alone is generally not sufficient for the development of RMS. **(B)** Survival analysis of *Pax3:Foxo1a,p53*; *Pax3:Foxo1a,Rb1*, and *p53,Rb1* mice (all using *Myf6cre*). The results suggest that *Rb1* loss, unlike *p53* inactivation, has no cooperative effects with Pax3:Foxo1a activation for the development of RMS. **(C)** Addition of *Rb1* inactivation to *Pax3:Foxo1a* activation and *p53* deletion significantly accelerated rhabdomyosarcomagenesis compared with *Pax3:Foxo1a,p53* mice (log-rank test, *P* <0.01; all experiments using *Myf6cre*). The mean latency for sarcoma development was 67 days in *Pax3:Foxo1a,p53,Rb1* mice and 107 days in *Pax3:Foxo1a,p53* mice. **(D)** Tumor stage (top) and anatomical site (bottom) of mice corresponding to **(A)** to **(C)**.

To investigate the role of *Rb1* in aRMS, we restricted our conditional model studies to the *Myf6* lineage using *Myf6cre* on the basis of our prior studies indicating the maturing myoblast to be the likely aRMS cell of origin [2]. *Rb1* homozygous deletion in the *Myf6Cre* lineage can lead to pituitary macroadenomas [14], and therefore sarcoma-free survival (instead of tumor-free survival) is presented in Figure 3. We first inactivated both alleles of *Rb1* in *Myf6*-expressing maturing myofibers (designated hereafter as *Rb1* mice). Animals were born in normal Mendelian ratios and developed normally throughout adolescence and early adulthood (Figure 3A). As reported previously [2], for mice with only *Pax3:Foxo1a* homozygous activation or only *p53* homozygous inactivation (*Pax3:Foxo1a* or *p53* mice, respectively), aRMS occurred but at very low frequency (Figure 3A). Also as reported previously, simultaneously inactivating *p53* dramatically increased the frequency and decreased the latency of aRMS tumors in *Pax3:Foxo1a*-expressing mice [2]. However, *Rb1* loss had no cooperative effect on the tumor development with either *Pax3:Foxo1a* activation or with *p53* inactivation (Figure 3B). Interestingly, when *Rb1* loss was combined with *Pax3:Foxo1a* activation and *p53* inactivation concurrently, the overall latency of tumor formation decreased (Figure 3B). Taken together, these data suggested that *Rb1* loss is a modifier of disease progression – but not a necessary and sufficient mutational event, nor a strong cooperative initiating mutation.

**Figure 4:**
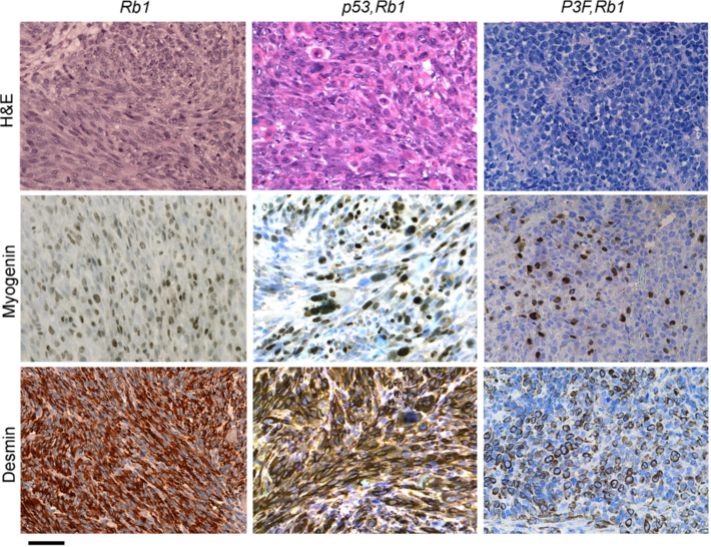
**Histological analysis of rare *Rb1 *and *Pax3:Foxo1a,Rb1 *tumors.** A representative *Rb1* tumor (left) shows spindle cell morphology with high percentage of myogenin-positive and desmin-positive cells consistent with eRMS, whereas a representative *Pax3:Foxo1a,Rb1* tumor (right) consists of small round blue cells that are only rarely myogenin and desmin positive (best region shown in figure), consistent with the diagnosis of poorly differentiated malignant epithelioid neoplasm. Scale bar: 40 μm. H&E, hematoxylin and eosin.

**Figure 5:**
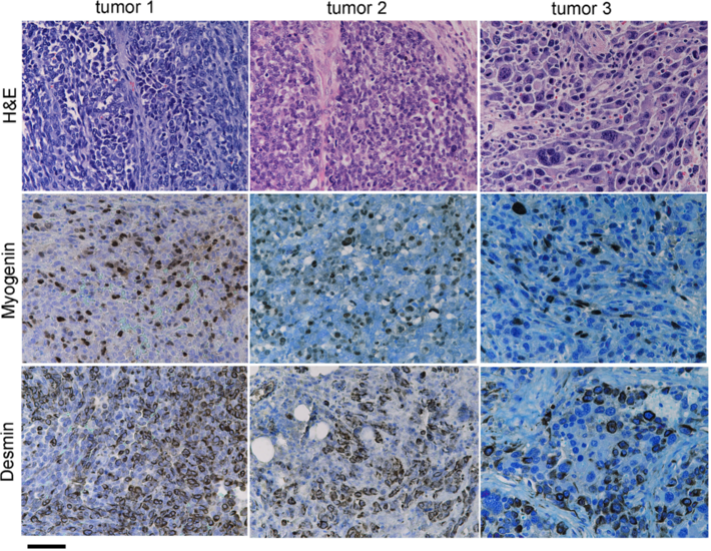
**Histological analysis of *Pax3:Foxo1a,p53,Rb1 *tumors.** Histological analysis of *Pax3:Foxo1a,p53,Rb1* tumors demonstrate small round blue cells with positive myogenin and desmin staining, consistent with aRMS (tumor 1 and tumor 2). However, some tumors showed highly anaplastic morphology (tumor 3) and were diagnosed as pleomorphic RMS. The *Pax3:Foxo1a* transcript level by qRT-PCR for the right tumor was nearly the same as for the middle tumor (data not shown). Scale bar: 40 μm. H&E, hematoxylin and eosin.

Figure 3C,D show the anatomical sites and tumor stages in each genetically engineered model. *Pax3:Foxo1a,p53,Rb1* mice demonstrated slightly more head/neck tumors and more large, nonmetastatic stage I tumors compared with *Pax3:Foxo1a,p53* tumors for which the *Rb1* locus was intact. Histologically*, Pax3:Foxo1a,Rb1* tumors consisted of myogenin and desmin-positive small round blue cells, consistent with the diagnosis of aRMS, whereas *Rb1* tumors were represented as mixed spindle and small round blue cells with only focal regions of myogenin or desmin positivity consistent with either RMS not otherwise specified or poorly differentiated malignant epithelioid neoplasms (Figure 4). Similarly, *p53,Rb1* tumors appeared as mixed spindle and small round blue cell histology with myogenin and desmin positivity and occasional rhabdomyoblasts, consistent with pleomorphic RMS (Figure 4). In contrast, *Pax3:Foxo1a,p53,Rb1* tumors sometimes retained histological identity as aRMS, but often had a mixed epithelioid/spindle cell morphology and variable myogenin and desmin staining (Figure 5). Pleomorphic histomorphology was present to varying degrees, often very extensive. When not consistent with aRMS, the spectrum of diagnoses included RMS not otherwise specified, pleomorphic RMS and undifferentiated spindle cell sarcoma.

### Addition of Rb1 inactivation to Pax3:Foxo1a activation and p53 deletion creates a bi-phenotypic profile using traditional aRMS and eRMS biomarkers

Since *Pax3:Foxo1a,p53* and *Pax3:Foxo1a,p53,Rb1* tumors had differences in histomorphology, we examined whether *Rb1* inactivation altered the expression level of *Pax3:Foxo1a*, thereby potentially altering expression of downstream target genes. Instead, *Pax3:Foxo1a,p53* and *Pax3:Foxo1a,p53,Rb1* tumors expressed the same level of *Pax3:Foxo1a* (Figure 1A). We also examined aRMS and eRMS-specific gene expression from tumors (Figure 1B). *Rb1* inactivation increased the expression of two markers, *Tcfap2* (*Transcription factor AP-2b*) and *Cdh3* (*Placental P-cadherin*), which have been identified as direct target genes of PAX3:FOXO1A in aRMS [14,15]. Paradoxically, *Pax3:Foxo1a,p53,Rb1* tumor also showed an increased level of *Hmga2* (*Transcription factor high mobility group A*), a marker of fusion-negative aRMS [15]. The expression level of *EGFR* (*Epidermal Growth Factor Receptor*) and *Fbn2* (*Fibrillin-2*) as specific markers for eRMS [16,17] were also paradoxically increased in *Pax3:Foxo1a,p53,Rb1* tumors. Furthermore, *Pax3:Foxo1a,p53,Rb1* tumors also had increased expression of *Myogenin*, a marker for alveolar and embryonic rhabdomyoblastic differentiation [18], compared with *Pax3:Foxo1a,p53* tumors. These results suggested that *Rb1* inactivation in the context of *Pax3:Foxo1a* activation and *p53* inactivation may mix the molecular phenotype of tumors for a state neither consistent purely with aRMS or with eRMS.

### Rb1 loss in Pax3:Foxo1a,p53 tumors results in an overall molecular phenotype more similar to aRMS than eRMS

**Figure 6:**
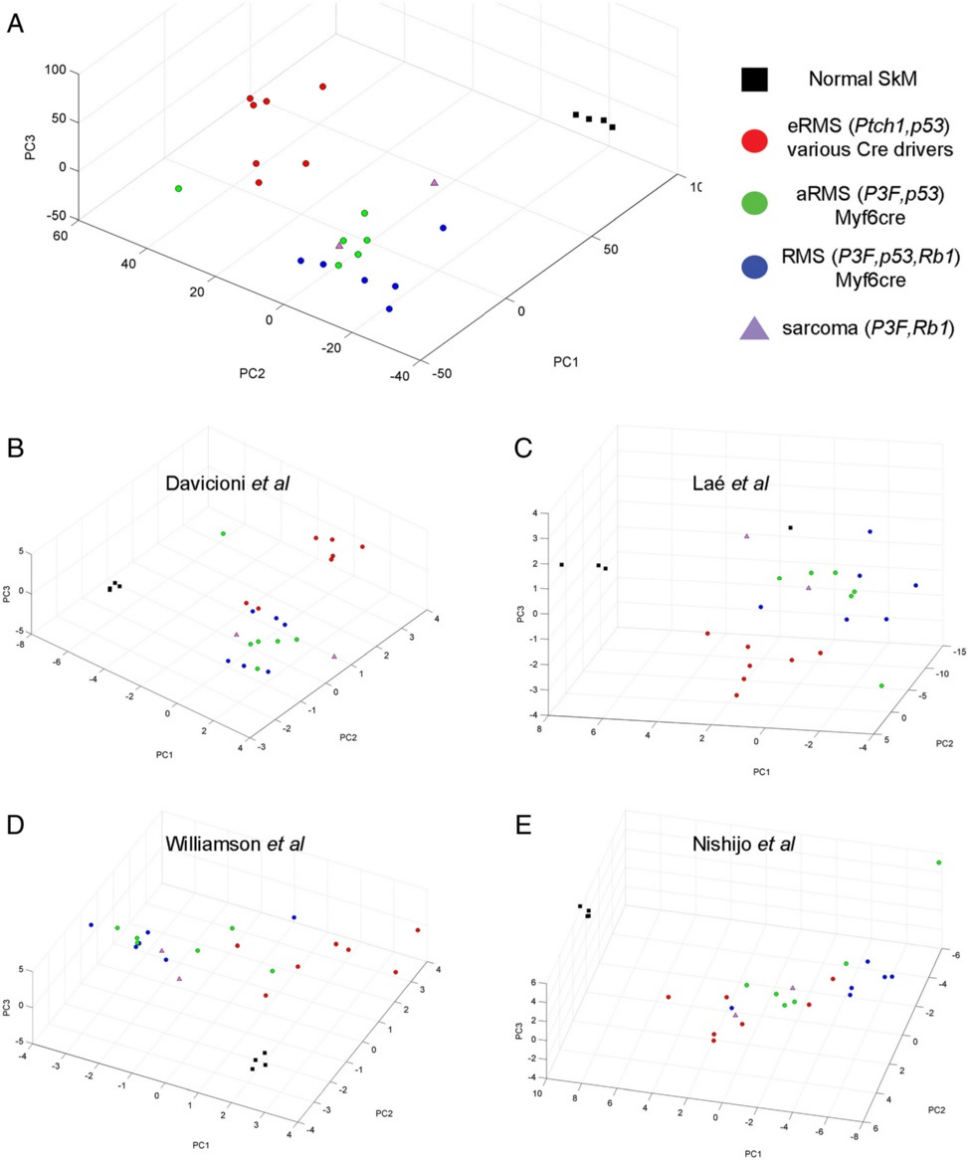
**Human aRMS versus eRMS gene set differences are conserved in murine models when *Rb1 *is inactivated. (A)** Principal component analysis (PCA) of mouse tumors using 12,370 genes that significantly discriminate among previously described murine eRMS [1] (red), *Pax3:Foxo1a,p53* tumors (green) or *Pax3:Foxo1a,p53,Rb1* tumors (blue), and normal skeletal muscle (black). Samples are colored according to their genotypes as indicated. Average log_2_ intensity >6 and standard deviation >0.1 over 25 samples. Normal skeletal muscle (SkM) is shown as a control. Error bars represent the standard error of the mean. **(B)** PCA for differentially expressed genes between PAX-FOXO1A and fusion-negative RMS cell lines by Davicioni and colleagues [14]. **(C)** PCA for differentially expressed genes in fusion-positive aRMSaRMS versus fusion-negative eRMS by Laé and colleagues [19]. **(D)** PCA for differentially expressed F1/F2 metagenes by Williamson and colleagues [20]. **(E)** PCA for genes conserved in mouse and human Pax3:Foxo1a-positive aRMS by Nishijo and colleagues [11].

Because the addition of *Rb1* loss sometimes masked histological identity and also shifted selected marker expression of aRMS versus eRMS for *Pax3:Foxo1a,p53* mice, we sought to clarify overall biology of *Pax3:Foxo1a,p53,Rb1* mice by examining global gene expression profiles. To achieve this goal, we applied PCA to all 25 tumor samples [GEO:GSE22520] [1] with 12,370 selected probes based on their overall expression level and variation, as well as published four gene sets that differentiated aRMS and eRMS in humans [11,14,19,20] (see Methods). All PCA results derived from four different gene sets showed comparable separation of three groups: eRMS (red), aRMS (green) and normal skeletal muscle (black) (Figure 6A to E). In addition, we observed that *Pax3:Foxo1a,p53* tumors (green), *Pax3:Foxo1a,p53,Rb1* tumors (blue) and *Pax3:Foxo1a,Rb1* tumors (purple) were classified into the same relative RMS types (Figure 6A to E). The genes and principal component coefficients (that is, loadings) for genes are given in Additional file 1. As a validation measure, the recombination of *Rb1* loci from tumors was confirmed to be complete in *Pax3:Foxo1a,p53,Rb1* tumors (Additional file 2). Furthermore, we performed a Student *t* test between *Pax3:Foxo1a,p53* tumor (aRMS) and *Pax3:Foxo1a,p53,Rb1* tumor (other RMS) data with 138 genes differentially expressed between these two groups (fold-change >2, and *P <* 0.05). Classical genes recognized for *Rb1*-deficient tumors [21] were identified as increased in *Rb1* deleted aRMS tumors (*Mcm7*, *H1fx*, *Cdc25c*, *Tyms*, *Brca2*, *Top2a*, *Kif2c*, *Tk1*, *Plk1*, *Birc5*, *Cdc20*, *Msh6*, *Cbx2*, *Chaf1b*, *Ccnb1*, *H2afz*, *Mcm2*) by 1.5-fold to 2.1-fold. In addition, intactness of the *Rb1* loci was associated with expression of certain myogenesis-related genes (*Myh7*, *Myl4*, *Actc1*, *Tnni1*, *Myl3*, *Mef2c*), whereas *Rb1* loss was associated with genes that did not fit any apparent common function (*Biklk*, *Itgb4*, *Slc14a1*, *Reln*, *Ear11*, *Vgll2*, *Pvalb*) (Additional file 3).

We next examined the functional and therapeutic significance of *Rb1* loss. pRb associates with a wide range of transcription factors to control cell cycle progression, cellular senescence, apoptosis, and differentiation. The best characterized role for pRb is in the control of E2F1 activity. pRb exerts this function by interfering with the ability of E2F1 to communicate with the basal transcription apparatus and/or recruiting chromatin-modifying enzymes to block the activation of E2F responsive genes [22]. In this context pRb has been shown to target histone deacetylase (HDAC) [23,24]. On the other hand, pRb is regulated by cyclin-dependent kinase (CDK)-4 or CDK6 in complex with cyclin D_1_[21,25,26] – rendering *Rb1 null* tumors insensitive to CDK4/CDK6 inhibitors. We therefore compared the sensitivity of primary tumor cell cultures from *Pax3:Foxo1a,p53* tumors with *Pax3:Foxo1a,p53,Rb1* tumors for the anti-cancer agents panobinostat (LBH58; a pan-HDAC inhibitor), PD0332991 (a selective cyclin D kinase 4/6 inhibitor), SAHA (vorinostat; a HDAC inhibitor) and SNS-032 (BMS-387032; a CDK2, CDK7 and CDK9 inhibitor). For this experiment, we utilized three biologically independent primary cell cultures for each genotype*.* We found no statistically significant difference in sensitivity to panobinostat at single concentrations (*P* = 0.38 at 10 nM, *P* = 0.34 at 20 nM and *P* = 0.28 at 40 nM; *P* values were based on analysis of variance tests with Bonferroni multiple testing corrections) (Figure 2A), but small and statistically significant trend differences were seen for panobinostat and PD0332991. No difference in sensitivity was seen for SAHA or SNS-032. These results suggested that *Pax3:Foxo1a,p53* tumors are functionally the same regardless of the deletion status of *Rb1*.

Given that pRb status has been previously shown to determine sensitivity to Cdk4/6 inhibitors in other forms of cancer [27], the insensitivity to PD0332991 for *Pax3:Foxo1a,p53,Rb1* tumors relative to *Pax3:Foxo1a,p53* tumors was unexpected. We thus hypothesized that aRMS with intact *Rb1* loci may nonetheless functionally inactivate pRb through epigenetic silencing or pRb hyperphosphorylation. To investigate these possibilities, we first examined the level of pRb and phospho-pRb by western blotting. We compared expression of Pax3:Foxo1a expressing primary tumor cell cultures with or without *Rb1* loss (*n* = 3 biological replicates each) to proliferating or differentiating C2C12 myoblasts as a control for the aRMS cell of origin. While present, pRb and phospho-pRb expression was dramatically lower in aRMS primary cell cultures for which *Rb1* alleles were wildtype than in C2C12 myoblasts (Figure 2B). As expected, pRb expression was absent in aRMS primary cell cultures for which *Rb1* was homozygously, conditionally deleted (Figure 2B). Expression of the Rb-related family member, p107, was not significantly increased in aRMS primary cell cultures for which *Rb1* was homozygously, conditionally deleted versus aRMS primary cell cultures for which *Rb1* alleles were wildtype (Figure 2B). Taken together, these data suggest that pRb expression is downregulated at the transcriptional or post-transcriptional level, thereby accounting for the lack of difference of sensitivity to the CDK4/CDK6 inhibitor, PD0332991, whether Pax3:Foxo1a-expressing tumors had wildtype or conditionally deleted *Rb1* alleles.

To determine whether decreased pRb levels in aRMS *Rb1* wildtype tumors reflected transcriptional downregulation, we performed qRT-PCR of *Rb1*. Relative to proliferating or differentiated C2C12 myoblasts, mRNA levels were significantly diminished in aRMS *Rb1* wildtype primary tumor cell cultures (Figure 2C). Given that *Rb1* was downregulated at the transcriptional level, to determine whether Pax3:Foxo1a acted directly or indirectly to reduce pRb expression we generated stable clones for knockdown of *Pax3:Foxo1a* using shRNA against eYFP (because the mouse model has *eYFP* expressed on the same mRNA as *Pax3:Foxo1a* by means of a *Pax3:Foxo1a-ires-eYFP* conditional knock-in configuration at the *Pax3* locus, *eYFP* knockdown leads to *Pax3:Foxo1a* knockdown). Despite reduction of Pax3:Foxo1a in two independent aRMS clones cultures relative to two independent control shRNA aRMS clone cultures, pRb expression did not change (Figure 2D). Furthermore, sensitivity to the CDK4/CDK6 inhibitor, PD0332991, was not improved by Pax3:Foxo1a knockdown (Figure 2E). These data suggest an alternation in G_1_/S checkpoint control in mouse aRMS that is independent of Pax3:Foxo1a.

**Figure 7:**
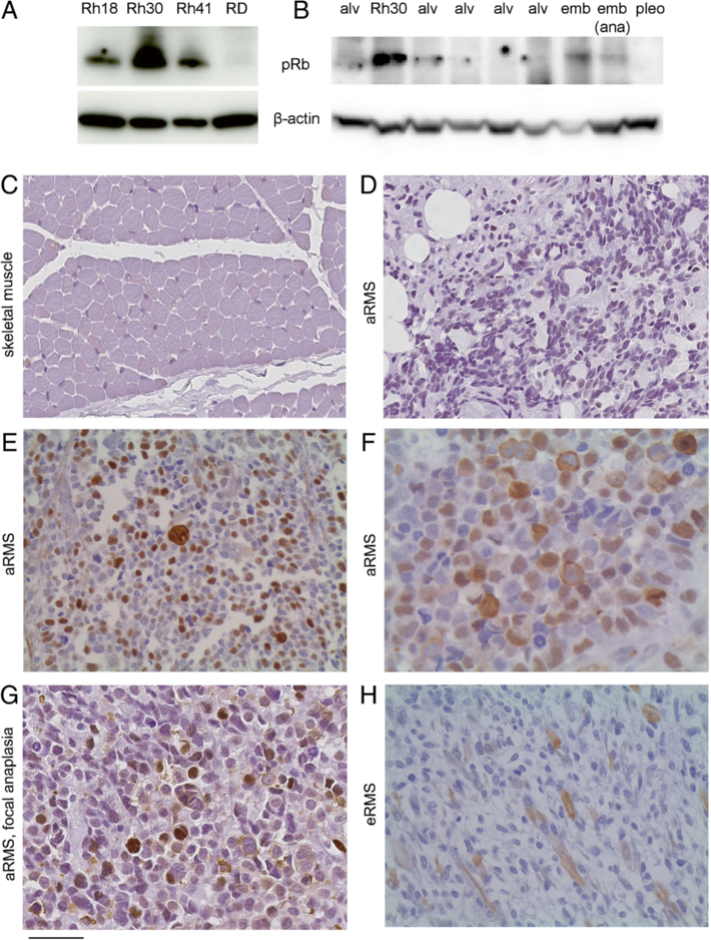
**pRb expression in human RMS. (A)** Western blotting of human aRMS (Rh30,Rh41) and eRMS (Rh18, RD) cell lines. **(B)** Clinical RMS samples. alv, aRMS; emb, eRMS; ana, with anaplasia; pleo, pleomorphic RMS. **(C)** to **(H)** Immunohistochemistry of a human rhabdomyosarcoma tissue microarray using an anti-phospho pRb antibody that detects phosphorylation at Ser807/811. Staining was nuclear, cytoplasmic or both in any given cell, but the percentage of cells stained variably between 0 and 80%.

To cross-correlate mouse aRMS findings to human pediatric aRMS, we examined pRb expression by western blotting in aRMS cell lines (Rh30, Rh41) in comparison with eRMS cell lines (Rh18, RD) (Figure 7A). Both aRMS cell lines expressed pRb, strongest in Rh30. To determine whether pRb expression in Rh30 was representative of clinical sample expression, we performed western blotting of available human aRMS, eRMS and pleomorphic RMS samples concurrent with Rh30 (Figure 7B; Additional file 4). Rh30 expression was an outlier, given that clinical aRMS (as well as eRMS and pleomorphic RMS) samples expressed little pRb.

To determine whether low pRb expression in RMS is due to homogeneous low pRb expression across all cells or selective pRb expression in only a subset of RMS cells, we performed immunohistochemistry of a tissue microarray provided by the Children’s Oncology Group Biorepository. This tissue microarray was evaluated using an anti-phospho-pRb antibody that detects phosphorylation at Ser807/811. Ser807 is a site phosphorylated by CDK4 that in some contexts appears critical to phospho-pRb growth suppressor function inactivation and nuclear export [28]. Results are presented in Additional file 5. Skeletal muscle consistently had no staining. For tumor cores with a typical aRMS histology, 3/25 (12%) had no expression, 12/25 (48%) had expression in 2 to 30% of cells, and 10/25 (40%) had weak to strong expression in 40 to 80% of cells. Nuclear expression was evident in 19/25 (76%) of cores, cytoplasmic expression in 11/25 (44%) of cores, and simultaneous nuclear and cytoplasmic expression was present in the same cell for 9/25 (36%) of cores. Altogether, 14/25 (56%) of aRMS cores displayed evidence of cytoplasmic phospho-pRb localization, suggesting that nuclear export may be a major mechanism of pRb inactivation in aRMS. In three other core samples of aRMS with anaplasia, 1 to 50% of cells strongly expressed phospho-pRb with nuclear localization (for so few samples we hesitate to infer any generalizations). Finally, for specialized rhabdomyoblast cells of aRMS that paradoxically express markers of differentiation and display common multinucleation but also express markers of proliferation (ki67 positivity) [29], phospho-pRb localization was nuclear, cytoplasmic or both (as was also seen for the nonrhabdomyoblast tumor cells). Expression of pRb was thus heterogeneous in aRMS, accounting for overall low total pRb levels – with high pRb expression levels in the Rh30 cell line possibly having been a selection effect.

## Discussion

In this study we have demonstrated that *Rb1* loss is a modifier of aRMS progression, but not a necessary and sufficient mutational event for rhabdomyosarcomagenesis, nor even a strong cooperative initiating mutation. The modifier effect of *Rb1* loss at the histological level was to increase anaplasia and pleomorphism, whereas at the molecular level, even though *Pax3:Foxo1a* expression itself was not altered, the traditional gene expression biomarkers of alveolar versus embryonal RMS subtypes were both increased. Individual gene expression biomarkers of eRMS versus aRMS may thus be unreliable in the situation of *Rb1* loss. Nevertheless, overall gene expression of *Rb1* null aRMS more closely approximated aRMS than eRMS. Intrinsically abnormal *Rb1* levels and pRb function in all Pax3:Foxo1-expressing RMS was evidenced by the insensitivity to a canonical Cdk4/6 inhibitor, regardless of whether the *Rb1* locus was intact or null. The mechanism of *Rb1* transcriptional dampening remains an open question for future studies. Although our testing of the HDAC1/2/3/6 inhibitor vorinostat had relatively little single agent effect on cell viability, it is intriguing to speculate that other pharmacological modifiers of DNA methylation, histone acetylation or histone methylation might restore *Rb1* levels and pRb function and thereby have utility in a combination therapy approach*.*

The role of *Rb1* in RMS initiation is controversial [6,7]. While RMS is rare as a primary cancer in patients with germline *Rb1* haploinsufficiency, RMS is the most common soft tissue sarcoma in a radiation field [30] for these patients. However, these cases are generally RMS not otherwise specified rather than aRMS [31]. In mice, the T antigen (which inactivates all three Rb-family members pRb, p107 and p130) expressed as a transgene leads to the development of cardiac RMS [32]. However, in our recent study of strict conditional *Rb1* loss in the *Myf6*-expressing fetal/postnatal maturing myoblast or Pax7-expressing postnatal muscle stem cell (satellite cell) lineages, no tumors developed [33]; instead, satellite cell and myoblast pools expanded but were largely incapable of fusing to form mature myofibers. Thus, from these past and the current studies it would seem that *Rb1* loss alone does not initiate rhabdomyosarcomagenesis.

A role for *Rb1* loss in progression of eRMS and other soft tissue sarcomas has been clearer than for aRMS. In a related report of non-aRMS soft tissue sarcomas, *Rb1* loss accelerated progression of *p53-*initiated tumors and led to undifferentiated phenotypes, but, as expected, did not induce tumor initiation in a conditional model using a *Prx-cre* driver (specific to the mesenchymal tissue of the limb bud) [34]. For RMS, *Rb1* had been suggested to play a more important role in embryonal RMS (eRMS) than aRMS: *Rb1* genetic abnormalities (allelic imbalance, deletion) are more common in eRMS than in aRMS [6], and one study showed no dramatic loss of *Rb1* in 13 aRMS primary tumor samples [7]. At the protein level, pRb positivity by immunohistochemistry in aRMS is lower than for eRMS (65% vs. 85%, respectively) [35]. Our complementary re-analysis of confirmed fusion-positive human aRMS revealed that a fully pRb off signature can be frequent (25 of 62 Pax3:Foxo1a-positive cases) but almost never does a fully pRb off signature happen without a co-existing p53 off signature (Jinu Abraham and colleagues, in preparation. Subtle variations in *Rb1* gene and protein expression may thus depend on the mutational profile of aRMS (for example, *Pax3:Foxo1a* and/or *p53* status) if not other factors. In the small sets of human samples we studied for total pRb expression by western and phospho-pRb expression by immunohistochemistry, we found that overall expression was generally low for aRMS tumors (similar to the mouse), and that only subsets of cells had expression within a tumor mass (and among this subset, cytoplasmic localization for presumed pRb inactivation was not uncommon).

An unexplained phenomenon is that human aRMS are known to have a much higher mitotic rate than eRMS [35], similar to the observation in mice [1]. A related observation in our current study was the fairly similar insensitivity of *Rb1* null and *Rb1* wildtype aRMS to a Cdk4/6 inhibitor, PD0332991, which may be attributed to the relatively low *Rb1* transcript levels we observed in tumors with wildtype *Rb1* alleles. We speculate that human aRMS tumors may achieve effective pRb inactivation (and related PD0332991 resistance) through the same or a number of other mechanisms including pRb nuclear exclusion, inhibition of pRb phosphatases, *Rb1* mutation, Survivin overexpression [27,36,37], *Cdk4* amplification [38], ΔNp73 or p57 expression [39], Cdkn2a (p16^ink4a^) loss, E2F gene mutations, overexpression or amplification of cyclin D_1_ (facilitating pRb phosphorylation), expression of viral proteins (for example, HPV-E7), or p27 or p21 loss [21]. The latter (p21 and p27) are observed to have lower expression in aRMS than eRMS, an effect that can be reversed by the putative HDAC inhibitor butyrate [40]. Further downstream in the G_1_/S checkpoint, p27 degradation is increased in a Pax3:Foxo1a-dependent manner, attributed to the Pax3:Foxo1a target gene product, Skp2 [11,41]. Interestingly, in other tumors p27 loss desensitizes *Rb1* null tumor cells to Arf-mediated apoptosis. Thus, p27 and pRb loss of function may be synergistically tumorigenic in aRMS – which combined with the other factors accelerating early G_1_/S checkpoint entry may overall accelerate progression from the G_1_ phase to the S phase.

An interesting aspect of our studies is that conditional deletion of *Rb1*, resulting in loss of the very low baseline expression of *Rb1* and pRb, could be associated with reduced myogenic marker expression for some tumors examined. pRb is known to have roles in both cell cycle control and myogenic differentiation of normal myoblasts, but when pRb is lost then p107 is able to play a compensatory role in myogenic differentiation [42]. In our studies of aRMS, p107 did not compensate for pRb loss. Thus, the variably present *Rb1* null aRMS de-differentiation phenotype suggests that low baseline pRb expression is in fact important biologically – and an important determinant of aRMS histomorphological identity. Diagnostically, this result could be very significant in that it leaves the possibility that some clinical cases of undifferentiated pleomorphic sarcomas may in fact express Pax3:Foxo1A, but in the context of pRb loss would not be tested for Pax3:FoxO1A given their histological appearance.

## Conclusions

The pRb and Pax3:Foxo1a status may warrant investigation in pleomorphic soft tissue sarcomas currently thought to be distinct from aRMS. A careful distinction, too, between low baseline pRb expression and near-complete pRb loss may require additional clinical biomarkers such as p16^ink4a^ in a prospective manner.

## Abbreviations

aRMS: Alveolar rhabdomyosarcoma; CDK: Cyclin-dependent kinase; DMEM: Dulbecco’s modified eagle’s media; eRMS: Embryonal rhabdomyosarcoma; HDAC: Histone deacetylase; PCA: Principal component analysis; RMS: Rhabdomyosarcoma; shRNA: Short hairpin RNA.

## Competing interests

The authors declare that they have no competing interests.

## Authors’ contributions

KN and CK conceived and designed these studies. Experimental methodology was developed by KN, KK and CK. Experimental procedures were carried out by KN, CK, MNS, AB, SD, SLP, PSM and ETH. ET, H-IHC, KK, ETH, KN, JA OR, MS, NB, YC, AM, BPR and CK participated in analysis and interpretation of data. CL, LAZ and JEM performed the statistical analysis. ET, KK and CK wrote the manuscript. All authors read and approved the final manuscript.

## Supplementary Material

Additional file 1: Table S1 Presenting gene sets used for PCA and genes accounting for each principal component in Figure 6. Click here for file

Additional file 2: Figure S1 Presenting complete recombination of floxed alleles. Successful recombination of all floxed alleles of *Pax3:Foxo1a, p53 or Rb1* was confirmed by genomic polymerase chain reaction of tumors in *Myf6cre,Pax3:Foxo1a,p53,Rb1* mice. Click here for file

Additional file 3: Table S2 Presenting differential gene expression for *Myf6cre,Pax3:Foxo1a,p53* tumors with and without *Rb1* inactivation. Click here for file

Additional file 4: Table S3 Presenting samples used for western blotting in Figure 7B. Click here for file

Additional file 5: Table S4 Presenting scoring for phospho-pRb of the tissue microarray (slide scan sent to the Children’s Oncology Group Biorepository and available on request). Click here for file
