# Supplementary figures and images for: Rb1 loss modifies but does not initiate alveolar rhabdomyosarcoma
Source: Skelet Muscle. 2013 Nov 25;3:27. doi: 10.1186/2044-5040-3-27 (PMC4177545; doi:10.1186/2044-5040-3-27)

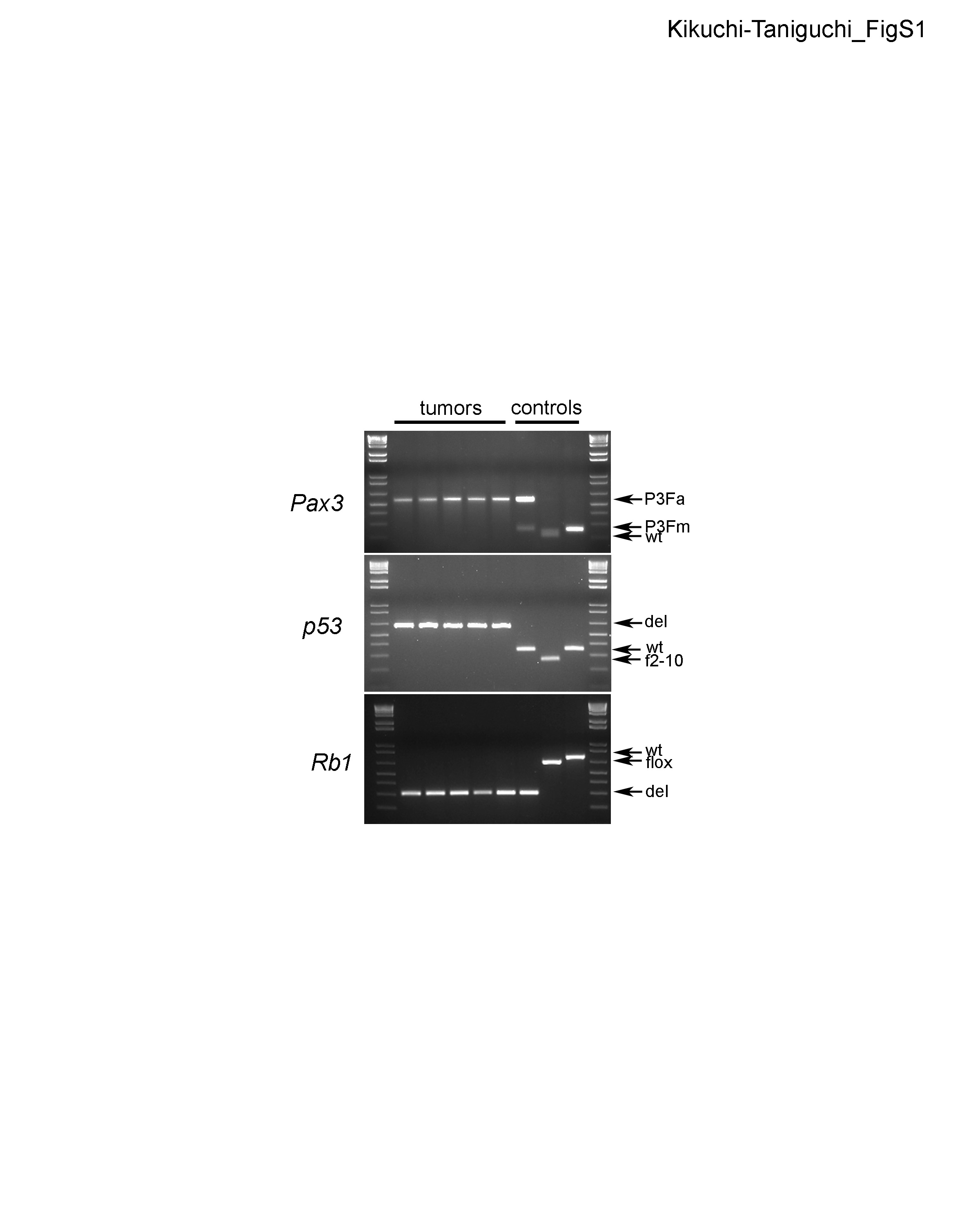

Supplement: Additional file 2: Figure S1 — Presenting complete recombination of floxed alleles. Successful recombination of all floxed alleles of Pax3:Foxo1a, p53 or Rb1 was confirmed by genomic polymerase chain reaction of tumors in Myf6cre,Pax3:Foxo1a,p53,Rb1 mice. [file 2044-5040-3-27-S2.jpeg]
